# Capacitance Determination for the Evaluation of Electrochemically Active Surface Area in a Catalyst Layer of NiFe-Layered Double Hydroxides for Anion Exchange Membrane Water Electrolyser

**DOI:** 10.3390/ma17030556

**Published:** 2024-01-24

**Authors:** Zhong Xie, Wei Qu, Elizabeth A. Fisher, Jason Fahlman, Koichiro Asazawa, Takao Hayashi, Hiroshi Shirataki, Hideaki Murase

**Affiliations:** 1 Energy, Mining and Environmental Research Centre, National Research Council of Canada, 4250 Wesbrook Mall, Vancouver, BC V6T 1W5, Canada; wei.qu@nrc-cnrc.gc.ca (W.Q.); elizabeth.fisher@nrc-cnrc.gc.ca (E.A.F.); jason.fahlman@nrc-cnrc.gc.ca (J.F.); 2 Applied Material Technology Center, Technology Division, Panasonic Holdings Corporation, 3-1-1, Yagumonakamachi, Moriguchi 570-8501, Osaka, Japan; asazawa.koichiro@jp.panasonic.com (K.A.); hayashi.takao@jp.panasonic.com (T.H.); shirataki.hiroshi@jp.panasonic.com (H.S.); murase.hideaki@jp.panasonic.com (H.M.)

**Keywords:** anion exchange membrane, H_2_ production, non-noble metal oxide catalysts, catalyst layer, electrochemically active surface area

## Abstract

The determination of the electrochemically active surface area (ECSA) of a catalyst layer (CL) of a non-precious metal catalyst is of fundamental importance in optimizing the design of a durable CL for anion exchange membrane (AEM) water electrolysis, but has yet to be developed. Traditional double layer capacitance (C_dl_), measured by cyclic voltammetry (CV), is not suitable for the estimation of the ECSA due to the nonconductive nature of Ni-based oxides and hydroxides in the non-Faradaic region. This paper analyses the applicability of electrochemical impedance spectroscopy (EIS) compared to CV in determining capacitances for the estimation of the ECSA of AEM-based CLs in an aqueous KOH electrolyte solution. A porous electrode transmission line (TML) model was employed to obtain the capacitance–voltage dependence from 1.0 V to 1.5 V at 20 mV intervals, covering both non-Faradic and Faradic regions. This allows for the identification of the contribution of a NiFe-layered double hydroxide (LDH) catalyst and supports in a CL, to capacitances in both non-Faradic and Faradic regions. A nearly constant double layer capacitance (Q_dl_) observed in the non-Faradic region represents the interfaces between catalyst supports and electrolytes. The capacitance determined in the Faradic region by EIS experiences a peak capacitance (Q_F_), which represents the maximum achievable ECSA in an AEMCL during reactions. The EIS method was additionally validated in durability testing. An approximate 30% loss of Q_F_ was noted while Q_dl_ remained unchanged following an eight-week test at 1 A/cm^2^ constant current density, implying that Q_F_, determined by EIS, is sensitive to and therefore suitable for assessing the loss of ECSA. This universal method can provide a reasonable estimate of catalyst utilization and enable the monitoring of catalyst degradation in CLs, in particular in liquid alkaline electrolyte water electrolysis systems.

## 1. Introduction

Electrochemical water splitting using renewable energies has long been recognized as a most promising clean and sustainable alternative for the production of green hydrogen. At present, commercial green hydrogen production employs alkaline water electrolysers (AWE) or proton exchange membrane (PEM)-based water electrolysers (WE). PEMWE requires expensive precious metal catalysts and Nafion-based membranes, resulting in high hydrogen production costs, and so cannot compete economically with fossil fuel-based hydrogen production. AEM water electrolysis is a promising alternative, having the potential to combine the advantages of both PEMWE and AWE to produce hydrogen [1] by using earth-abundant metal oxides or hydroxides as the anode oxygen evolution reaction (OER) catalysts. 

Numerous efforts have been devoted to developing various non-precious metal OER catalysts [2,3,4,5,6,7]. A number of transition metal oxides or hydroxides are reported to exhibit catalytic activity that approaches or even outperforms that of IrO_2_ catalysts for OER in ex-situ evaluations [8,9]. To advance the water splitting technology to commercial application, catalyst materials have to be integrated into actual electrodes and cells for performance evaluation. However, when integrated into CLs for OER, their catalytic performances are not readily reproduced [10] because catalytic performance is governed not only by catalyst composition, but also by the micro-environments in a CL. For effective assessment of their catalytic activities and catalyst utilization in a CL during water electrolysis, the determination of their ECSA in a CL becomes of paramount importance. Knowing the ECSAs and their changes in a CL will enable improvements in CL engineering, maximize catalyst utilization, and enhance the performance of a CL in AEMWE. 

For ECSA evaluation, several approaches [11], including the Brunauer–Emmett–Teller (BET), turnover frequency (TOF), CV, and EIS methods, have been presented [12], mostly relating to catalyst material development. The BET method has been used to determine the surface area of porous materials [13], but it cannot differentiate between electrochemically active and inactive surface sites [14]. Also, TOF has been proposed to quantify the in-situ active sites and stability of Ni−Fe (O_xy_) hydroxides for OER [15].

CV has been widely used for ECSA evaluation. For Pt-based catalysts and CLs, the ECSA measurement by CV has been well-developed [16]. Pt-based catalysts have well-defined hydrogen adsorption and desorption peaks, also called hydrogen under potential deposition (H-UPD) peaks, and CO oxidation profiles [17] in CV scanning, which can be used to calculate the coulombic charge for the ECSA based on the generally accepted specific charges of 210 μC/cm^2^ Pt for H-UPD or 420 μC/cm^2^ Pt for CO stripping [18]. 

For non-precious metal oxide or hydroxide catalysts, there are no such well-defined characteristic redox peaks like H-UPD or CO stripping peaks, which can be linked to their ECSA because no commonly agreed upon voltage window exists for redox wave/charge corresponding to changes in the oxidation state of the metal cations. Further, the specific coulombic charges for most metal cation oxidations are unavailable. A specific charge of 514 mC/cm_Ni_^2^ has been reported for the reduction peak of Ni(OH)_2_ (~0.05 V vs. a reversible hydrogen electrode (RHE)) [18], but this value is not equivalent to those of various Ni-based oxide or hydroxide catalysts with multiple oxidation states. Also uncertain is the reversibility of the redox peak. Dionigi et al. observed an irreversible oxidation cycle of CoFe-LDH in the first and subsequent CV cycles [19] because of the “charge-trapping” effect. The catalyst at the surface of CL, reduced by cathodic back scan, becomes less conductive because its conductivity depends on voltage. This prevents e-conduction to the “bulk” materials beneath the now less conductive surface layer [11]. In addition, it is a challenging task to identify the redox species of the peak and to obtain a corresponding specific charge due to the limited knowledge of surface redox reactions of metal cations [18]. Thus, using redox peaks in CV to estimate the ECSA of non-precious metal oxide or hydroxide-based CLs becomes difficult.

Instead, a non-Faradic double layer capacitance-based method has been proposed for the evaluation of metal oxides or hydroxides in catalyst material development. However, specific capacitances are not always available for non-precious metal oxides or hydroxides used in ECSA conversion [18]. For most, no widely accepted benchmarking specific capacitances exist, and those values reported by different groups vary widely. For example, metal oxide C_s_ values range from 22 μF/cm^2^ to 130 μF/cm^2^, with 40 μF/cm^2^ considered a universal for metal oxide surfaces in some studies [20], while others consider 60 μF/cm^2^ more realistic [21]. For Ni(Fe)O_x_H_y_ in the charge state, a C_s_ of ~80 μF/cm^2^ has been determined [11]. Variation in surface structure and composition renders uncertain such measured values of specific capacitances from metal oxides or hydroxides. Further affecting the C_s_ of most nickel-based oxides or hydroxides is the non-conductivity displayed in the non-Faradic region. Thus, the determination of the ECSA of non-precious metal oxide or hydroxide catalysts remains an unsolved but vitally important challenge [22].

EIS is another common method for measuring the ECSA of non-precious metal oxide or hydroxide catalysts in situ during catalyst material development. Watzele et al. employed EIS to determine the ECSA of NiO_x_ [22], MnO_x_ [14], and Ni-, Co-, Fe-, Ir-based, and Pt or Pt/Carbon electrocatalysts [23]. This method correlated the ECSA and the adsorption pseudocapacitance from reversibly adsorbed reaction intermediates. It assumes that adsorption pseudocapacitance originates only from the electrochemically active sites because the reaction occurs solely on the surface of the active material at the applied voltage. Singh et al. used EIS to determine the chemical capacitance for ECSA calculations in evaluating the intrinsic activity of metal−organic framework-based oxygen evolution electrocatalysts [24]. For OER, McCrory et al. used both EIS and CV in the non-Faradic region to obtain capacitances for ECSA estimation to benchmark the heterogeneous oxide electrocatalysts CoO_x_, CoP_i_, CoFeO_x_, NiO_x_, NiCeO_x_, NiCoO_x_, NiCuO_x_, NiFeO_x_, and NiLaO_x_ [20]. Connor et al. used a simplified Randles circuit with a constant phase element (CPE) to determine the capacitance for the ECSA in a MnO_x_ system [25]. For the estimation of the ECSA and intrinsic activity of NiFe-LDH, Jeon et al. investigated the use of EIS at reactive OER potentials to extract the capacitance that is hypothesized to arise due to reactive OER intermediates (O*, OH*, OOH*) adsorbed on the catalyst surface [26]. 

All efforts show that either EIS or CV or both can be utilized for capacitance determination and the ECSA estimation for OER catalyst materials (deposited film or nanoparticles) in aqueous solutions using a three-electrode system. However, to the best knowledge of the authors, there is no report regarding how reliable CV and EIS are for in-situ ECSA estimation in an AEM water electrolyser where catalyst materials are integrated into CLs within porous electrodes. The catalyst activity in CLs might differ greatly from that evaluated in solution electrolytes. The activities of catalysts integrated into CLs are governed not only by the catalyst itself but also by other factors, such as the binder/ionomer type, support material, microstructure of CLs, and electrolyte type and concentration. Other properties of the CL, such as hydrophilicity and hydrophobicity, pore structure and dimension, tortuosity, and porosity, also affect the accessibility of active sites and utilization of catalysts in the CL. In a porous electrode system, the capacitances become a combination of contributions from catalysts and supports (including PTL). Moreover, the catalytic active sites may become inaccessible at high current density due to gas evolution and subsequent mass transportation limitations caused by gas bubbles, complicating the ECSA determination in CLs. 

This paper examines the effectiveness of EIS compared to CV for capacitance determination using a CL fabricated with NiFe-LDH electrocatalysts as the sample CL for an AEMWE with an alkaline electrolyte. Although the analysis was conducted on an example CL, the methodologies can be applicable to characterizing other types of CL having non-precious metal oxide or hydroxide catalysts in liquid-based electrolytes, enabling the optimization of CL fabrication and CL durability studies.

## 2. Experimental Setup and Procedure

**Figure 1:**
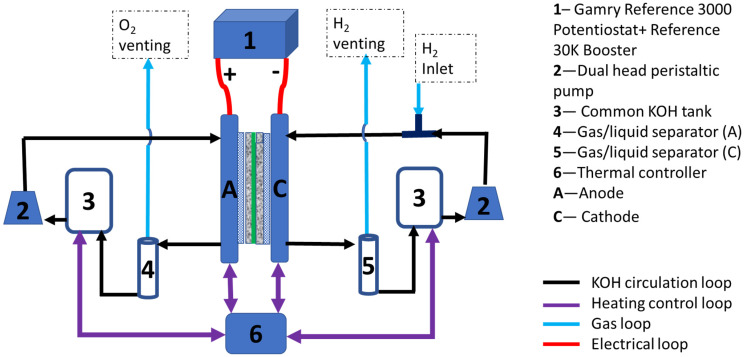
Schematic of the AEM water electrolyser cell testing system.

Anode OER CLs were fabricated with custom-made NiFe-LDH supported by nickel particles, which have shown superior performance both in ex- and in-situ evaluations [9], at a loading of 1.7 mg/cm^2^. The cathode used a traditional Pt/C catalyst for the hydrogen evolution reaction, at a loading of 1.8 mg/cm^2^. A 25 cm^2^ membrane–electrode assembly (MEA) was tested using an inhouse-made single cell jig with straight flow channels in Nickel-200 plates. Figure 1 shows the MEA testing system schematically. 

The system uses a dual head peristaltic pump to feed solution from a common 1 M KOH electrolyte reservoir (20 L capacity) into the anode and cathode chambers separately. The liquid/gas streams exhausting from the anode and cathode outlets flow through their respective gas/liquid separators. The liquid electrolytes from the gas/liquid separators flow back to the KOH reservoir. The gases vent into separate O_2_ and H_2_ exhaust systems. The flow rate is set at 10 mL/min for both the anode and cathode. A 5 mL/min H_2_ stream is injected into the cathode KOH electrolyte stream through a Swagelok T fitting, which then enters the cathode of the cell to serve as a reversible hydrogen reference electrode (RHE) during CV and EIS measurements (All voltages in this work are referred to RHE). The temperature of the cell was controlled at 80 °C. 

A Gamry Reference 3000 Potentiostat/Galvanostat (Gamry Instruments, Warminster, PA, USA) equipped with a Reference 30K Booster was used for CV scanning and impedance measurements. The CV scanning voltages were set to range from 0.9 to 1.1 V in the cathode containing 1 M KOH electrolyte solution in the non-Faradic region at scan rates of 5, 10, 25, 50, 100, 200, 400, and 800 mV/s. The cell rested under open circuit voltage (OCV) for 5 min between each CV scanning. 

EIS measurements were run in voltage-controlled mode with an AC signal frequency in the range of 100 kHz to 1 Hz from 1.0 to 1.5 V in 20 mV increments. These voltages were selected to cover both the non-Faradic and Faradic regions, including the possible redox of surface metal cations, adsorption of OER intermediate species, and OER charge transfer. The cell rested at OCV for 5 min between each EIS measurement. The AC signal amplitude was set at 10 mV RMS for voltages from 1.0 to 1.3 V and 20 mV RMS for voltages above 1.3 V.

## 3. Theoretical Considerations

### 3.1. Capacitance and ECSA

The basis of ECSA evaluation is capacitance (C) measurement. Assuming that the electrochemical capacitance per unit of surface area is uniformly distributed across the CL, the ECSA of a CL will be proportional to C

ECSA = C/C_s_ (1)

where C_s_ is the specific area capacitance of the sample catalyst or is the capacitance of an atomically smooth planar surface of the material per unit area under identical electrolyte conditions [20]. 

However, real systems like AEM porous electrodes do not show purely capacitive behaviour, but instead a CPE due to capacitive and resistive elements distributed along the porous electrode structure. In this case, the physical capacitor is presented by a CPE, which is expressed in complex notion in Equation (2) with parameter Q of unit F/s ^1−α^ [20],

(2) $$Z_{CPE}=\frac{1}{{\left(j\omega \right)}^{\alpha }Q}$$

where α is the phase angle, *j* is the imaginary unit, and *ω* is angular frequency.

For 0 < α < 1, Q does not represent a simple capacitance, but is proportional to the ECSA [27]. When using Q as a measure of the ECSA of the electrode, the Q parameter is obtained by fitting the capacitive branch of the Nyquist plot.

Once having obtained a Q value, the ECSA of a CL can be estimated according to Equation (3):

ECSA = Q/C_s_ (3)

Correlation between the ECSA of the electrode and the capacitance requires knowing the value of C_s_ for the catalyst materials. However, there is no universally accepted standard specific capacitance for individual non-precious metal oxide or hydroxide catalysts. For NiFe-LDH, a C_s_ of 300 μF/cm^2^ has been reported as a reference for ex-situ characterization [19,26,28]. To find a proper C_s_ value to assess the catalyst active surface or catalyst utilization after the catalysts have been incorporated into a CL is even more challenging. However, for AEMCL characterization, if the purpose is to compare the degradation of a CL or of catalyst utilization during CL fabrication, the C or Q value alone will be sufficient. This paper focuses on C and Q determination rather than on conversion to ECSA due to the paucity of accurate C_s_ values.

### 3.2. Determination of Capacitance for a Porous Electrode

The capacitances are determined using two methods: (1) by measuring the non-Faradic capacitive current associated with double-layer charging from the scan-rate dependence of cyclic voltammograms, and (2) by measuring the frequency-dependent impedance of the system using EIS. 

#### 3.2.1. Determination of the Capacitance of a Porous Electrode by CV

The measurement of double-layer charging of electrochemical capacitance via CV is a well-known technique [29]. A voltage range where no apparent Faradic processes occur is selected. The current measured in this non-Faradic region is assumed to be due to double-layer charging or discharging. The charge current and discharge current are then measured from CV at multiple scan rates. The double layer capacitance (C_dl_) is given by Equation (4).

(4) $$C_{dl}=\frac{i_{c}}{v}$$

where

i _c_ is the charging current and *ν* is the scan rate.

Thus, a plot of i _c_ as a function of *ν* yields a straight line with slope equal to C_dl_, which links with the ECSA of a CL by Equation (1).

#### 3.2.2. Determination of the Capacitance of a Porous Electrode by EIS

**Figure 2:**
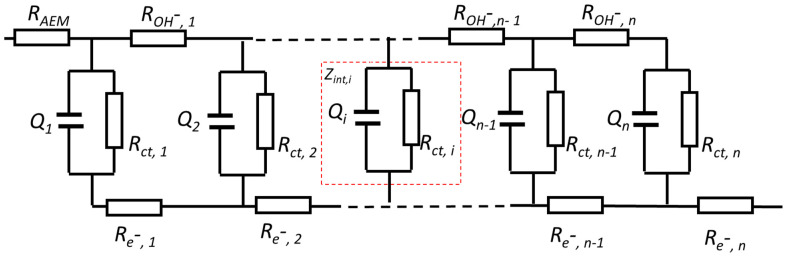
Typical porous TML model for AEMCL.

When non-precious oxide or hydroxide catalyst particles are integrated into a CL as the AEM water electrolyser electrode, porous electrode theory [30] and a TML model can be used to simulate the ECSA. There are many TML equivalent circuit models that can represent the impedance of the porous CL [31,32,33,34,35]. A typical TML model for OH^−^ anion conductive CL is depicted in Figure 2.

The AEM anode CL can be viewed as a porous electrode and treated as a parallel array of uniform cylindrical pores having uniformly distributed electrolyte resistance, R_OH_^−^, and specific interfacial impedance, Z_int,i_ (depicted within the red dotted square in Figure 2), between the electronic and ionic phases [33]. R_ct,i_ and Q_i_ represent interfacial charge transfer resistance and double layer capacitance distributed within the porous CL. In a CL made with nickel-supported transition metal catalysts, the resistance R_e_- of the e-conduction rail can be considered to be negligible. As such, de Levie pore electrode theory can then be used to describe the impedance response [36].

For simplicity, all the pores are considered to be identical in a homogeneous CL. The CL can then be represented as a collection of one-dimensional homogeneous cylindrical pores connected in parallel, having average representative parameters [34]. For the OER in the anode at small currents, the impedance of the CL, Z_CL_, can be written as Equation (5) [31,37]

(5) $$Z_{CL}=\sqrt{Z_{int}R_{{OH}^{-}}}Coth\sqrt{\frac{R_{{OH}^{-}}}{Z_{int}}}$$

where

(6) $$Z_{int}=\frac{R_{ct}}{1+{\left(j\omega \right)}^{\alpha }QR_{ct}}=1/\{\frac{1}{R_{ct}}+(j\omega {)}^{\alpha }Q\}$$

R_OH_^−^ is the ionic resistance in the pores within the CL, Z_int_ is the total interfacial impedance in the pores within the CL, and R_ct_ is the charge-transfer resistance. 

In a region with non-Faradic reaction or no metal cation redox, the impedance is obtained by assuming infinite charge-transfer resistance, R_ct_, in the CL. When R_ct_ approaches infinity, Equation (6) simplifies:

(7) $$Z_{int}=\frac{1}{Q({j\omega )}^{a}}$$

From the measured Z_CL_ in both non-Faradic and low polarization Faradic regions, Q can be obtained for assessing capacitances, linked to the ECSA through Equation (2). Q represents the capacitance determined by EIS and differs from the double layer capacitance C_dl_ determined by CV in this paper. 

Instead of looking for a numerical solution for the complex Equation (5), which has been analysed at length in references [31,32], the focus of this paper is to fit experimental impedance data from an AEM single cell to determine the capacitance Q for ECSA evaluation.

In our experimental setup, the cathode impedance can be neglected when H_2_ is used as RHE in the KOH electrolyte feed loop. The Warburg diffusion element, *W_s_*, can also be ignored in the non-Faradic and low polarization regions. A de Levie TML equivalent circuit model is used to fit the Z_CL_ for the determination of capacitance Q and other components of the CL under different conditions. 

## 4. Experimental Results and Discussion

### 4.1. Capacitance (Q) Determination Using EIS

**Figure 3:**
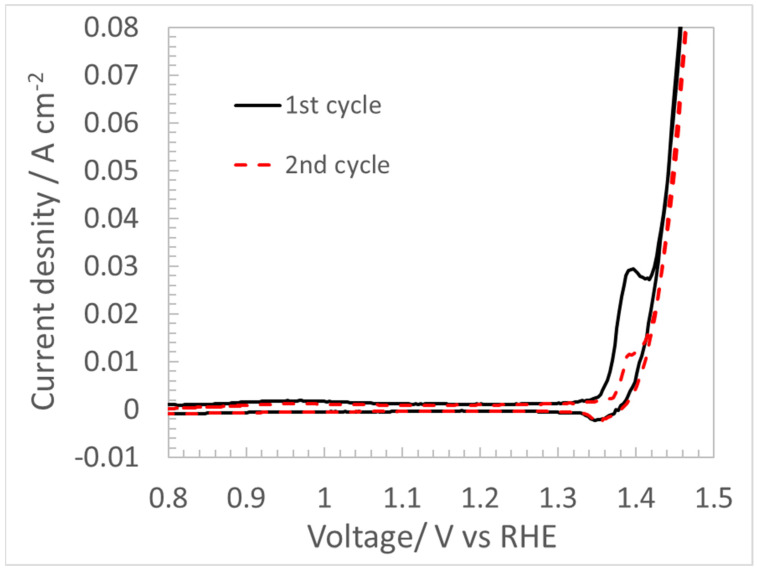
CV profiles of AEMCL from 0.8 to 1.5 V with a scan rate of 5 mV/s in 1 M KOH electrolyte feed stream at 80 °C.

First, a CV scan at 5 mV/s was carried out to understand the polarization behaviours of the NiFe-LDH catalyst layer in 1 M KOH. As shown in Figure 3, nearly flat charge/discharge regions are observed at less than 1.30 V. Irreversible metal cation redox peaks, which shrank in the subsequent scanning, are observed in CV curves in the Faradic region near 1.39 V. The decreasing redox peaks in the second CV cycle are ascribed to the “charge-trapping” effect as observed by Dionigi et al. [19]. The catalyst at the surface of the CL becomes less conductive during the cathodic back scan. This prevents e-conduction to the “bulk” materials beneath the now less conductive surface layer [11]. Subsequently, the amount of available Ni^3+^/Ni^2+^ redox species is reduced.

**Figure 4:**
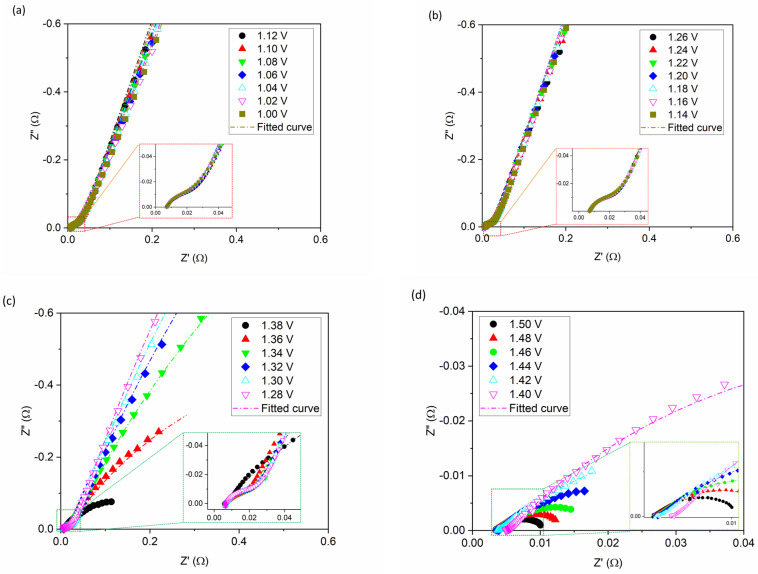
Anodic EIS responses with 20 mV increments for 25 cm^2^ AEM-MEA in 1 M KOH, 80 °C from (**a**) 1.0 to 1.12 V, (**b**) 1.14 to 1.26 V, (**c**) 1.28 to 1.38 V, and (**d**) 1.40 to 1.50 V. Marker dots represent the data collected. The dashed lines are the fitted curves using the model shown in Figure 2.

The EIS measurements are scanned from 1.0 to 1.5 V in 20 mV increments, covering both the non-Faradic (1.0 to ~1.3 V) and Faradic regions (~1.3 to 1.5 V). Typical EIS responses of the cathode are shown in Figure 4.

**Figure 5:**
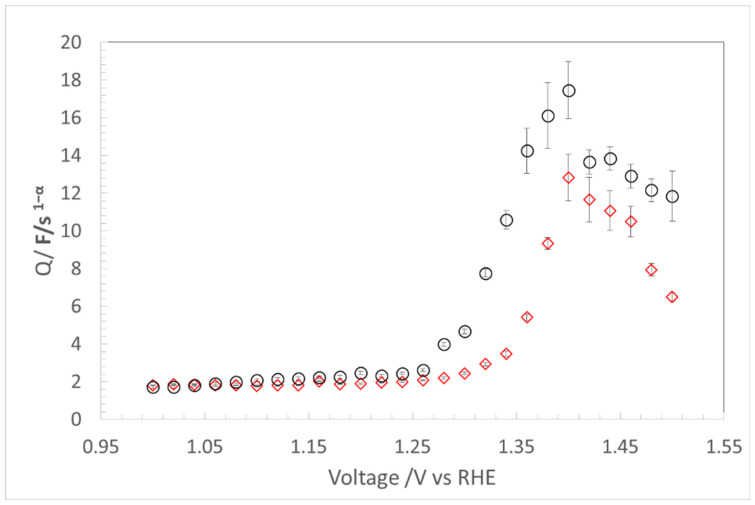
Total capacitance Q_t_ determined by EIS with de Levies TML model against voltage from 1.0 to 1.5 V in 1 M KOH at 80 °C. -○- represents the beginning of life, and -◊- after eight-week testing at 1 A/cm^2^.

The TML model fits the data well from the non-Faradic region to the Faradic region, but the error bars for fitting in the Faradic region are slightly larger. The total capacitance (Q_t_) values and corresponding error bars obtained by EIS fitting are plotted against voltage in Figure 5. 

The Q-voltage profile in Figure 5 shows a nearly constant Q value of ~2 F/s ^1−α^ from 1.0 to 1.26 V. Q increases with voltage rising above onset voltage of OER, at approximately 1.26 V. In the Faradic region, Q reaches a peak value at approximately 1.4 V. 

#### 4.1.1. Non-Faradic Region

**Figure 6:**
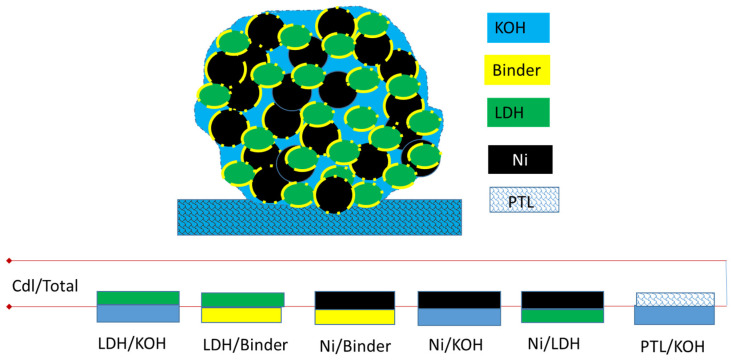
Illustration of capacitance structure from different interfaces in a CL.

In the non-Faradic region, the CL behaves as an electrical double layer capacitor. Contributions to capacitance are from interfaces of various components in the CL, and are illustrated in Figure 6.

In AEMCLs, ion conduction is dominated by OH^−^ ions in the pores filled with aqueous KOH electrolyte. The interfaces between the aqueous KOH/NiFe-LDH catalyst and liquid KOH/Ni particles or other conductive supports, such as NiFe-LDH and PTL are the major contributors to electrical double layer capacitance. The Nafion ionomer functions only as the binder, to improve the hydrophilicity of CLs, and do not contribute to ion conduction. Where the AEM ionomer was used as the binder, the contribution of ionomer/support or ionomer/catalyst interfaces to capacitance can be considered as negligible due to a difference of two orders of magnitude in conductivity between 1 M KOH (~200 mS/cm) and the AEM ionomer (~1 mS/cm). The contribution of solid/solid interfaces to capacitance is also treated as negligible. Therefore, in the non-Faradic region, the measured capacitance represents the contribution of all of the interfaces of conductive sites accessible to KOH electrolyte ions. 

As reported [26], the NiFe-LDH is nonconductive before the onset voltage in the non-Faradic region, and, as such, will barely contribute to the ECSA. Therefore, the nearly constant Q_dl_ value of 2 F/s^1−α^ obtained in the non-Faradic region mainly represents the contribution of Ni support and PTL. 

#### 4.1.2. Faradic Region

Faradic processes, either redox of surface metal cations or interfacial electrochemical reactions or both, can only originate in the electrically conductive sites/interfaces [14]. Therefore, a connection between Faradic-induced capacitance and active surface area is more credible than one based on the double layer capacitance obtained in the non-Faradic region. 

As shown in Figure 4c,d, above 1.26 V, the EIS responses curve down rapidly from being nearly linear, becoming arcs. In Figure 5, above 1.26 V, the Q increases rapidly and then declines after peaking at approximately 1.40 V. The rapid increase of total capacitance Q_t_ with electrode polarization is related to the phenomenon that LDH conductivity is voltage dependant and becomes more conductive as voltage increases [28]. However, when the voltage continues to increase to above 1.4 V, the bubbles generated by the OER attach to active catalyst sites within the CL and begin to block the active sites, causing the Q_t_ values to start declining. Ideally, a plateau could be the best indicator of maximum capacitance available for ECSA calculation. However, because it is impossible to eliminate bubbles in an operating cell, obtaining a flat Q_t_ value from EIS voltage scanning is difficult. Consequently, under actual operating conditions peak Q_t_ is taken as the maximum attainable capacitance for a fabricated CL. 

Since Faradic capacitance and double layer capacitance both contribute to the total capacitance Q_t_ obtained by EIS, the net Faradic capacitance Q_F_ can be deduced by Equation (8) [38].

Q_F_ = Q_t_ − Q_dl_ (8)

While Q_dl_ is linked to the interfaces of catalyst supports/KOH, the net Q_F_ can be ascribed to the contribution of NiFe-LDH catalyst/KOH interfaces to the ECSA, assuming that the Q_dl_ remains constant in both Faradic and non-Faradic regions. 

Although peak Q_F_ does not actually represent the ECSA of a CL because of the bubble effect, Q_F_ can be used to estimate the maximum attainable ECSA for an AEM CL under actual operating conditions. Better design of CLs may effectively remove the bubbles from the CL, achieving higher Q_t_ and ECSAs. 

As can be seen from Figure 5, the peak Q_F_ of 15.47 F/s^1−α^ is nearly eight times the double layer capacitance Q_dl_ (~2 F/s^1−α^) of the non-Faradic region. Wei et al. reported the capacitance is to be up to three orders of magnitude greater in the high conductivity region than in the lower conductivity regions [18]. In this context, the Q_dl_ from the non-Faradic regions will misrepresent the actual ECSA of a CL. Q_F_ is truly more representative of the ECSA for an operating AEMCL. 

A similar approach exists that links Faradic pseudocapacitance of reaction intermediates to the ECSA in catalyst material development [14,22,23,26]. At 1.6 V, the adsorption process might overlap with charge transfer. Using EIS to distinguish between OER intermediate adsorbates and charge transfer is difficult in an operating CL.

### 4.2. CL Degradation Evaluation with Q_F_

A major obstacle to employing OER electrocatalysts and electrodes in large-scale practical applications is long-term operational stability. This problem becomes even more severe when the oxygen evolution electrocatalysts are operated at large current densities, e.g., 1000 mA/cm^2^, a practical value in water splitting. Factors such as, membrane failure, catalyst deactivation or loss, and oxidation of conductive supports, contribute to the degradation of an AEMCL. Decoupling the contribution of catalysts from that of supports to the degradation will provide important information for the improvement of CL design and optimization. Here, EIS was used to extract the C_dl_ and Q_F_ of the CL after an eight-week test at 1 A/cm^2^ current density. The results are presented in Figure 5, which shows at 1.4 V, the Q_F_ decreasing from a peak of 15.47 F/s ^1−α^ to 10.83 F/s ^1−α^, around a 30% reduction. But, the Q_dl_ remains almost unchanged at ~2.0 F/s^1−α^. We believe that Q_F_ determined by EIS is sensitive to the loss of catalyst activity or the ECSA during the durability test. The almost unchanged Q_dl_ indicates that Ni support/electrolyte interfaces were quite stable during the eight-week evaluation. Durability testing verifies that capacitance–voltage scanning by EIS is an effective method of differentiating between catalyst degradation and support change. Analysis of the catalyst degradation mechanism is beyond the scope of this paper.

### 4.3. CV for Double Layer Capacitance

**Figure 7:**
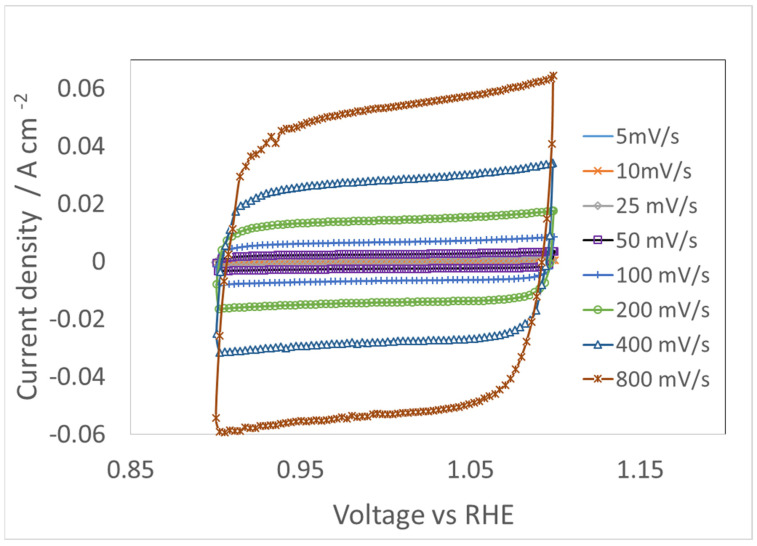
Cyclic voltammograms at different scanning rates for AEMCL in 1 M N_2_-purged KOH electrolyte at 80 °C.

For the AEMCL anode, a voltage window from 0.9 to 1.1 V was selected for measuring CV, using different scan rates in N_2_-purged KOH electrolyte. To better understand the difference between CV and EIS for double layer capacitance determination in the non-Faradic region, the method was extended in a study of the dependence of C_dl_ and Q_dl_ on ion concentration. Three different KOH concentrations (1 M, 0.5 M, and 0.1 M) were used. The CV scanning curves in 1 M KOH are presented in Figure 7. The CV results from the other two concentrations are similar and so are not shown here.

**Figure 8:**
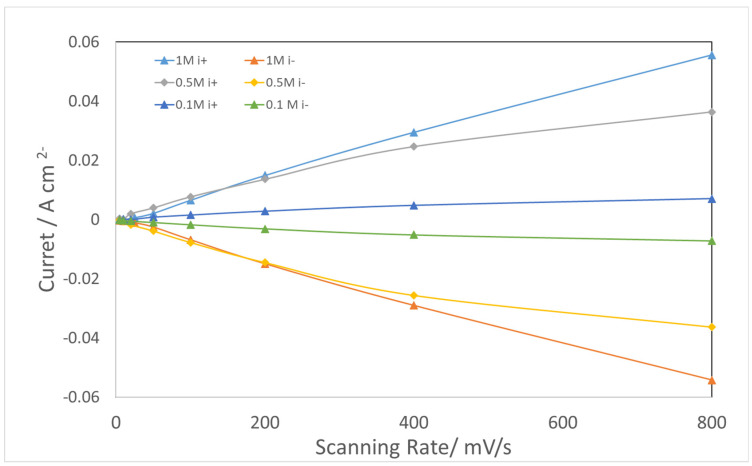
Double layer charge /discharge current vs. scanning rate plots for 1 M, 0.5 M, and 0.1 M KOH electrolyte.

The measured charge current density, $i_{+}$ , and discharge current density, $i_{-}$ , at 1.0 V for each scanning rate were selected to calculate the capacitances. The $i_{+/-}$ vs. scanning rate is plotted in Figure 8. The linear fitting of $i_{+/-}$ vs. the scanning rate produces slopes defined as C_dl_. A double layer capacitance of 1.9 F was calculated for the AEMCL in 1 M N_2_-purged KOH electrolyte.

Figure 8 shows the decline in C_dl_ with reduced KOH concentrations. The C_dl_ calculated for different KOH concentrations are listed in Table 1. For comparison, double layer capacitances extracted by EIS in the non-Faradic region at 1.0 V are shown in Table 1.

Using CV, the difference in results between 1 M KOH and 0.5 M KOH was not significant, but a considerable C_dl_ reduction was observed for 0.1 M KOH electrolyte. Weber et al. reported a declining trend of C_dl_ and ECSA, with the ECSA in water being one fifth of that in 1 M KOH [39]. This suggests that as the electrolyte concentration decreases, ion conduction within the pore structure of the CL becomes limited. Consequently, increasing ohmic loss begins to distort the CV profiles, so errors in C_dl_ determined by CV scanning increase as KOH concentration decreases. 

Table 1 shows that at 1.0 V, for 1 M KOH and 0.5 M KOH, the C_dl_ obtained from CV and the Q_dl_ determined by EIS are comparable. This indicates that both EIS and CV provide a good Q_dl_ or C_dl_ estimate for a CL in highly conductive ion solutions such as 1 M and 0.5 M KOH. However, for the lower concentration of 0.1 M KOH, the Q_dl_ obtained from EIS does not decrease as much as does the C_dl_ from CV. Upon comparison, we posit that in the non-Faradic region, EIS is simpler and superior to CV in double layer capacitance determination and catalyst layer support evaluation. 

## 5. Conclusions

The complex nature of the non-precious catalysts in CLs hinder accurate quantification of active sites and the associated ECSA. Double layer capacitances derived from the non-Faradaic region do not reliably represent the ECSA of a CL because the NiFe-LDH catalyst is nonconductive before OER onset voltage. Instead, double layer capacitance values are governed by the interfaces of catalyst support/electrolytes. Since only electrically active sites (interfaces) contribute to Faradic processes, it will be more logical to correlate Faradic capacitance Q_F_ from the Faradic region to the ECSA for a NiFe-LDH CL in an operational AEM water electrolyser. 

Using EIS, a capacitance-voltage profile to derive Q_dl_ and Q_F_ is obtained. A nearly constant Q_dl_ of ~2 F/s^1−α^ obtained in the non-Faradic region below the OER onset voltage around 1.26 V represents the availability of conductive interfaces of catalyst supports. The Q_F_ in the Faradic region increases rapidly because the voltage-dependant conductivity’s enhancement of LDH, reaching a peak value of 15.47 F/s^1−α^ at 1.4 V. It then declines with further increases in voltage because OER bubbles are generated, which block the active sites. Ideally, a plateau in Q-V profile would best represent the maximum ECSA of a CL, but seems impractical to attain in an actual operating AEMWE cell. We grant that the peak value might not represent the true ECSA, impeded as it is by the bubble effect, but the peak Q_F_ from the Faradic region yields the best obtainable estimation of the ECSA for a non-precious metal CL under operating conditions. Absolute ECSA values cannot be obtained directly due to lack of standard references (C_s_) for the capacitance of individual catalyst materials. 

The peak Q_F_ determined by EIS was used to assess the degradation of the AEMCL. While Q_dl_ remains almost unchanged, the peak Q_F_ value declines from 15.47 F/s ^1−α^ to 10.83 F/s^1−α^, an approximately 30% reduction after eight weeks of durability testing at 1 A/cm^2^ constant current density. Although analysis of the catalyst degradation mechanism is beyond the scope of this paper, results indicate that the Q_F_ determined by EIS is viable for assessing the loss of ECSA in the investigation of durability. The nearly constant Q_dl_ implies that catalyst supports (Ni particles and PTL) were stable during the eight-week degradation evaluation. Durability testing validates that capacitance-voltage scanning by EIS is a good mean of distinguishing catalyst loss from support degradation. 

EIS for Q_dl_ is less sensitive to electrolyte concentration than is CV. This implies that EIS is superior to CV for double layer capacitance determination in the non-Faradic region and for stability evaluation of catalyst supports, especially in systems with low alkaline concentration. 

## Figures and Tables

**Table 1:** Comparison of double layer capacitances obtained by CV with those of EIS at 1.0 V.

KOH Concentration/M	1	0.5	0.1
C_dl_ Charge/F	1.9	1.5	0.3
C_dl_ discharge/F	1.9	1.6	0.3
Q_dl_/F/s ^1−α^ from EIS @1.0 V	1.89	1.73	1.66

## Data Availability

The data presented in this study are available on request from the corresponding author.

## Nomenclature

AC	alternating current
AEM	anion exchange membrane
BET	Brunauer–Emmett–Teller
C	general definition of capacitance
C_dl_	double layer capacitance determined by CV method
CL	catalyst layer
CPE	constant phase element
C_s_	specific area capacitance
CV	cyclic voltammetry
ECSA	electrochemically active surface area
EIS	electrochemical impedance spectroscopy
H-UPD	hydrogen under potential deposition
i _c_	charging current density
*i_+_*	positive charge current density
*i_−_*	negative discharge current density
*j*	imaginary unit
KOH	potassium hydroxide
LDH	layered double hydroxide
MEA	membrane-electrode-assembly
OCV	open circuit voltage
OER	oxygen evolution reaction
PEM	proton exchange membrane
PTL	porous transport layer
Q	capacitance determined by EIS
Q_i_	double layer capacitance distributed within the porous CL
Q_t_	Total capacitance determined by EIS
Q_dl_	double layer capacitance determined by the EIS method in the non-Faradic region
Q_F_	Faradic capacitance determined by the EIS method in the Faradic region
R_ct,i_	interfacial charge transfer distributed within the porous CL
R_ct_	charge transfer resistance
R_e_,_i_	interfacial electron resistance distributed within the porous CL
RHE	reversible hydrogen electrode
RMS	root mean square
R_OH_^−^	ionic resistance in the pore within the CL
TOF	turnover frequency
TML	transmission line
ν	scanning rate
WE	water electrolysers
*W_s_*	Warburg diffusion element
Z_CL_	impedance of the CL
Z_int,*i*_	interfacial impedance for the unit length pore
Z_int_	total interfacial impedance in the pores within the CL
Z*_cpe_*	impedance of the CPE
α	impedance phase angle
ω	angular frequency
